# Identification of Hydroxyproline-Containing Proteins and Hydroxylation of Proline Residues in Rice

**DOI:** 10.3389/fpls.2020.01207

**Published:** 2020-08-07

**Authors:** Ronghong Liang, Li You, Fang Dong, Xiaolu Zhao, Jie Zhao

**Affiliations:** State Key Laboratory of Hybrid Rice, College of Life Sciences, Wuhan University, Wuhan, China

**Keywords:** hydroxyproline-containing proteins, hydroxyproline, Arabinogalactan proteins, glycomodule, rice

## Abstract

The hydroxyproline-containing proteins (HCPs) among secretory and vacuolar proteins play important roles in growth and development of higher plants. Many hydroxyproline-rich glycoproteins (HRGPs), including Arabinogalactan proteins (AGPs), extensins (EXTs), and proline-rich proteins (PRPs), are identified as HCPs by bioinformatics approaches. The experimental evidence for validation of novel proline hydroxylation sites is vital for understanding their functional roles. In this study, the 62 HCPs containing 114 hydroxyproline (O, Hyp) residues were identified, and it was found that hydroxylation of proline residues in the HCPs could either constitute attachment sites for glycans or have other biological function in rice. The glycomodules of AO, OA, OG, VO, LO, and OE were abundant in the 62 HCPs. Further analysis showed that the 22 of 62 HCPs contained both signal peptides and transmembrane domains, and the 19 HCPs only contained transmembrane domains, while 21 HCPs contained neither. This study indicated the feasibility of mass spectrometry-based proteomics combined with bioinformatics approaches for the large-scale characterization of Hyp sites from complex protein digest mixtures. Furthermore, the expression of AGPs in rice was detected by using β-GlcY reagent and JIM13 antibody. The results displayed that the AGPs were widely distributed in different tissues and organs of rice, especially expressed highly in lateral root, pollen and embryo. In conclusion, our study revealed that the HCPs and Hyp residues in rice were ubiquitous and that these Hyps could be candidates for linking to glycans, which laid the foundation for further studying the functions of HCPs and hydroxylation of proline residues in rice.

## Introduction

Glycosylation of proteins is widespread in various organisms. There are two main types of glycosylation, N-glycosylation and O-glycosylation. The N-glycosylation is usually bound to asparagine residues and has been studied in depth, while the O-glycosylation is more complicated. The O-glycosylation binds to serine and threonine residues in yeasts, fungi and animals and also to hydroxyproline (Hyp) residues in plants (Shimizu et al., 2005). Most studies of hydroxyproline-containing proteins (HCPs) are focused on the family of hydroxyproline-rich glycoproteins (HRGPs). HRGPs are separated into three families, Arabinogalactan proteins (AGPs), extensins (EXTs), and proline-rich proteins (PRPs) (Johnson et al., 2017). The HRGPs ranged from highly glycosylated AGPs to moderately glycosylated EXTs and minimally glycosylated PRPs in plant kingdom (Johnson et al., 2017). The AGPs usually contain 3 domains, N-terminal signal peptide, proline/hydroxyproline-rich domain, and C-terminal signal of glycosylphophatidylinositol (GPI) anchor (Schultz et al., 2002; Johnson et al., 2003; Pereira et al., 2014), which are necessary for their biological functions. The AGPs family can be divided into the classical AGPs (including lysine-rich classical AGPs), the non-classical AGPs, the AG peptides, and the chimeric AGPs. The chimeric AGPs with different domains are classified into fasciclin-like AGPs (FLAs), phytocyanin-like AGPs (PAGs), xylogen-like AGPs (XYLPs), and other chimeric AGPs (Ma et al., 2017).

The studies of AGPs in different developmental processes of plants involve Yariv phenylglycosides [1,3,5-tri (p-glycosyloxyphenylazo) 2,4,6-trihydroxybenzene] and antibodies that bind to AGPs specifically (Yariv et al., 1967). The Yariv reagent, especially β-glucosyl Yariv phenylglycoside (β-GlcY), provides an effective approach for the research of AGPs as histochemical and precipitated reagent (Sun et al., 2005; Seifert and Roberts, 2007). Further research indicates that Yariv phenylglycosides could be considered as specific binding reagents for β-1,3-galactan chains of AGPs (Kitazawa et al., 2013). In the field of plant research, there are few studies on the roles of glycoproteins and glycosylation. Most types of carbohydrates attaching to the AGP protein backbones were type II Arabinogalactan polysaccharides, which are covalently bound to clustered non-contiguous Hyps (Kieliszewski and Lamport, 1994; Kieliszewski et al., 1995). Mass spectrometry techniques have also been used to identify the Hyps in HCPs. In addition, AGP-specific mAbs (monoclonal antibodies), including JIM8 (Pennell et al., 1991), JIM13 (Knox et al., 1991), MAC207 (Pennell et al., 1989), and LM2 (Smallwood et al., 1996), are usually used to detect the expression of AGPs.

The amino acids of PAST (P, proline; A, alanine; S, serine; T, threonine) are rich in the AGPs of higher plants. For example, the classical AGPs contain at least 50% PAST (Ma and Zhao, 2010). Correspondingly, the AGPs contain characteristic glycomodules, including AO, OA, SO, TO, VO, and GO (Johnson et al., 2017). It was supposed that noncontiguous proline residues are hydroxylated and glycosylated on the basis of AGPs glycosylation (Liu et al., 2013). However, it is unclear that whether the proline residues in AGPs identified by bioinformatics were hydroxylated and glycosylated. In addition, the EXTs and PRPs belonged to HRGPs, and contained SPPP or SPPPP amino acid repeats and PVKCYT, KKPCPP, or PPVX (K/T) motifs, respectively (Johnson et al., 2017).

AGPs play very important roles in different aspects of plant growth and development, which is verified in the research of *Arabidopsis* Lys-rich classical AGPs. In the Lys-rich *AtAGP17* gene mutant, the efficiency of *Agrobacterium* transformation is significantly decreased (Gaspar et al., 2004). However, another Lys-rich AGP, AtAGP18, exerts an active regulation over the selection and survival of megaspores (Yang and Showalter, 2007; Zhang et al., 2011a; Zhang et al., 2011b; Demesa-Arevalo and Vielle-Calzada, 2013). Different from AtAGP17 and AtAGP18, the AtAGP19 is essential for cell division and expansion (Yang et al., 2007; Yang et al., 2011). Two Lys-rich AGPs, OsAGP12 and OsAGP13, are also identified in rice, but there are no related functional studies (Ma and Zhao, 2010).

Previous studies have found that AGPs function in the anther development of plants. *Arabidopsis* FLA3 (fasciclin-like AGP3) is involved in microspore development and pollen intine formation by participating in cellulose deposition (Li et al., 2010). Similar to the *atfla3* mutant, there is also seed abortion in the *atagp6* and *atagp11* double mutant plants (Levitin et al., 2008; Coimbra et al., 2009; Coimbra et al., 2010). Unlike AtAGP6 and AtAGP11 proteins, the orthologous BcMF8/18 (*Brassica campestris* male fertility 8/18) are involved in microspore development through independent pathways (Lin et al., 2018). Further study indicates that AtTEK (transposable element silencing *via* AT-hook) regulates *AGP6*/*11*/*23*/*40* genes expression in anthers to control nexine layer formation. Moreover, it is also proposed that glycoproteins might be essential components of the nexine layer in the pollen wall (Jia et al., 2015), therefore glycosyltransferases can be important for anthers development. The study of Ms8 (maize male sterile 8), a putative β-1,3-galactosyltransferase, showed that it modulates cell division, expansion, and differentiation during early anther development (Wang et al., 2013). The homologous protein of AtAGP23 in rice, *Oryza sativa indica* Arabinogalactan protein (OSIAGP), plays a role in seedling and pollen tube growth by regulating cell elongation (Anand and Tyagi, 2010). However, there is only a handful of research on AGPs in rice compared with *Arabidopsis*.

The studies have shown that AGPs are essential for plant cell wall development. In *Arabidopsis*, APAP1 (Arabinoxylan pectin AGP1) affects plant wall architecture involved in the sugar composition (Tan et al., 2013). In cotton, fasciclin-like AGP1 (FLA1) may function in cell wall matrix, thereby affecting fiber initiation and elongation (Huang et al., 2013). The previous study also showed that O-glycosylation on EXTs is essential for cell wall self-assembly and root hair elongation in *Arabidopsis* (Velasquez et al., 2011). These proteins are mainly expressed in the cell wall, and the GPI anchor sequences of HRGPs are usually essential for the subcellular localization.

In recent years, a large number of glycoprotein genes expressed in various tissues have been identified and classified by bioinformatics analysis in *Arabidopsis* and rice (Ma and Zhao, 2010; Li and Wu, 2012; Ma et al., 2014; Ma et al., 2017), but there were still lack of identification of hydroxyproline sites. In the study, the 62 HCPs containing 114 Hyp residues were identified in rice and the characteristic glycomodules were found by mass spectrometry technique, and the glycomodules of AO, OA, OG, VO, LO, and OE were rich in the these HCPs. The 22 of 62 HCPs contained both signal peptides and transmembrane domains. Therefore, it was feasible to extensively identify the modifications of oxidation, which provided the effective information for the study of rice proteins in the future. Furthermore, the research revealed the wide expression of AGPs in different tissues and organs of rice, which indicated the involvement of AGPs in various tissue-specific functions. These study revealed that the HCPs and Hyp residues in rice were ubiquitous, which provided new information for further studying the functions of HCPs and hydroxylation of proline residues in rice.

## Materials and Methods

### Plant Materials and Growth Conditions

The rice (*Oryza sativa L. japonica* cv. *Nipponbare*) plants were grown in a greenhouse of Wuhan University at 28°C to 32°C with a 16-h light and 8-h dark cycle. The rice materials for extracting glycoproteins were shoots and roots at 14 DAG (days after germination), panicles at P1 to P6 (P1: 0–3 cm, P2: 3–5 cm, P3: 5–10 cm, P4: 10–15 cm, P5: 15–22 cm, P6: 22–30 cm length), ovaries at 1 to 10 days after pollination (DAP), anthers and pistils at the mature stage.

### Extraction and Deglycosylation of AGPs

Extraction of AGPs was carried out by the modified Schultz’s method (Schultz et al., 2000). There were two independent biological replicates of each tissue and organ. The plant materials (50 g of roots, shoots, and panicles; 25 g ovaries; 10 g anthers and pistils, respectively) were ground to fine powder in liquid nitrogen. The ground materials were added to equal volume extraction buffer (50 mM Tris-HCl, 10 mM EDTA, 0.1% β-mercaptoethanol, and 1% Triton X-100, pH 8.0) and incubated at 4°C for 3 h. The samples were centrifuged for 15 min at 12,000 rpm, and the supernatant was precipitated with five volumes of ethanol at 4°C for overnight. The precipitate was re-suspended by vortex mixing in 5 mL of 50 mM Tris-HCl (pH 8.0), the insoluble precipitate was removed by centrifugation, and the supernatant was retained. The supernatant was freeze-dried overnight to concentrate the re-suspended solution. The dried samples were re-suspended in 250 to 500 μl of 1% NaCl and respectively transferred to 1.5 mL microcentrifuge tubes. AGPs were precipitated with the β-glucosyl Yariv reagent (β-GlcY) (Gane et al., 1995) by mixing the re-suspended samples in an equal volume of β-GlcY (2 mg/mL) in 1% NaCl and incubating overnight at 4°C. The insoluble β-GlcY-AGPs complex was collected by centrifugation at 12,000 rpm in a microcentrifuge for 1 h. The surplus β-GlcY reagent was removed by washing the pellet three times in 1% NaCl and then twice in methanol. The pellet was dried and dissolved in a minimum volume of dimethyl sulfoxide, and mixed with solid sodium dithionite. Double distilled water was added with mixing until the mixture became a clear yellow color. Then, the yellow solution was desalted using dialysis membrane (3500 Da) equilibrated with PBS (phosphate buffer solution), and the eluate was freeze-dried. Finally, the isolated glycoproteins were deglycosylated by the TFMS (trifluoromethanesulfonic) acid method using the Glyco-Profile IV kit (Sigma), and desalted by dialysis membrane (3500 Da) and stored at −80°C. Since the samples for rice mature anthers and pistils were too little, the glycoproteins were not deglycosylated.

### Identification of HCPs by Mass Spectrometry

The deglycosylated proteins were separated with 10% SDS-polyacrylamide (SDS-PAGE) gels (10% Acrylamide, 0.375 M Tris-HCl pH 8.8, 0.1% SDS, 0.1% ammonium persulfate, 0.06% TEMED) by electrophoresis to carry out mass spectrometry for analyzing proteins. When the samples containing bromophenol blue migrated into separating gel, electrophoresis was continued for 5 to 10 min until the bromophenol blue migrated 1 cm into the separating gel. About 1 cm SDS-PAGE gel without protein staining was cut into 1 mm^3^ cubes, and proteins were digested with trypsin (Taoka et al., 2003). LC-MS/MS data were obtained on a Q Exactive HF mass spectrometer system (Thermo Scientific, San Jose, CA, USA). The instrument was set in positive ion mode with a capillary temperature 275°C and spray voltage 2.4 kV acquisition was operated in data-dependent mode. The full scans were performed in the Orbitrap with a resolution set to 120,000 at 200 m/z. The full scan AGC target was 3×106 with a maximum injection time 20 ms, and the mass range was set to 300 to 1,800. MS/MS scans were operated in the HCD mode with normalized collision energy 27. The 20 most intense ions were selected for MS/MS acquisition in the Orbitrap with a resolution of 60,000. The AGC target for MS/MS scans was 1×106, with a maximum ion injection time 105 ms and intensity threshold 2.2×104, the isolation window was 1.6 Th, the fixed first mass was 100, and the dynamic exclusion time was 60 s. Tryptic peptides were dissolved in 0.1% formic acid and separated using an EASY-nLC 1000 system (Thermo Scientific, Odense, Denmark). Firstly, the peptides were loaded onto a trap column (Acclaim PepMapR 100, 100 μm×2 cm, C18, 5 μm, 100 A) at a flow rate of 3.0 μl/min, then subsequently eluted from the trap column onto an analytical column (Acclaim PepMapR RSLC, 75 μm × 25 cm, C18, 2 μm, 100 A) at a flow rate of 250 nL/min with a 60 min gradient.

The OsAGPs were obtained from the OsAGPs Database (Ma et al., 2017) and the new OsHCPs were obtained from UniProt Knowledgebase (UniProtKB) of *Oryza sativa subsp. japonica rice* (https://www.uniprot.org/uniprot/) by using software of Thermo Proteome Discoverer 2.1.0.8.1. The proteomic data were submitted into iProX (http://www.iprox.org) (Ma J. et al., 2019) and the project ID is IPX0001999000.

### KEGG Pathway Cluster Analysis

The 62 HCPs identified from rice were converted into suitable gene ID with the function of “Gene ID Conversion” in Bioinformatics Resources 6.8 (https://david.ncifcrf.gov/) (Huang et al., 2009a; Huang et al., 2009b). The genes were analyzed for KEGG pathway cluster with the function of “Gene Functional Classification” in Bioinformatics Resources 6.8.

### Semi-Thin Section of Tissues and Organs

The rice tissues and organs were quickly placed in a glass bottle with fixation buffer containing 4% paraformaldehyde and 1% glutaraldehyde in PBS, extracted air in vacuum for 0.5 to 1 h, and replaced with fresh fixation solution. In the next day, the samples were washed 5 times with PBS, dehydrated in different concentrations of ethanol, and then transferred to a different concentration of Technovit 7100 embedding agent and ethanol mixed solution. The samples were incubated 2 h at room temperature and extracted air in vacuum for 5 min. The samples were sequentially transferred to different concentrations of Technovit 7100 embedding agent solution (Liu et al., 2013) for polymerizing overnight at 4°C. They were polymerized at room temperature for 2 h and overnight at 37°C. The sections were sliced by semi-thin slicer (MT-X, USA) and placed on the treated glass slides with distilled water and dried at 37°C for further experimentation. At least three independent biological and three technical replicates were made.

### Immunoenzyme Localization of AGPs

The sections were washed in PBS, placed into the mixed digestive solution, incubated for 10 min at room temperature, washed with distilled water, and then incubated with 5 μl JIM13 antibody overnight at 4°C. They were incubated with goat anti-rat IgG at 37°C for 20 min, washed with PBS 5 times for 5 min each time, incubated in SABC reagent at 37°C for 20 min, and washed with PBS 5 times again. Finally, the sections were observed and photographed under a microscope (OLYMPUS, U-PMTVC, Japan) with a charge-coupled device CoolSNAP CCD (Photometrics, USA). At least three independent biological and three technical replicates were made.

For overall immunoenzyme localization, the samples were fixed in fixative (4% paraformaldehyde, 1% glutaraldehyde phosphate buffer, pH 7.0) at room temperature for 5 h or 4°C overnight and washed twice with distilled water or PBS. Subsequently, they were treated with 3% H_2_O_2_ at room temperature for 15 min, washed with distilled water 3 times for 5 min each time, and blocked with 5% bovine serum albumin (BSA) (Ma T. F. et al., 2019). At least three independent biological and three technical replicates were made.

### Staining Tissues and Organs With β-Glucosyl Yariv Reagent

The samples were placed in 4% paraformaldehyde solution for 5 to 7 h at room temperature to further investigate the expression of AGPs in various tissues and organs of rice. After the samples were washed three times with double distilled water, they were stained with 100 μM β-GlcY reagent for 6 to 7 h at room temperature or overnight at 4°C. They were washed three times with double distilled water again, and observed and photographed under an inverted microscope (OLYMPUS, IX-70). At least three independent biological and three technical replicates were made.

## Results

### Extraction of Glycoproteins From Different Tissues and Organs of Rice

Samples of shoots and roots at 14 DAG, P1-P6 panicles, 1 to 10 DAP seeds, mature anthers and pistils were collected to extract cell wall proteins of rice. The glycoproteins in the different tissues and organs were precipitated with β-GlcY reagent by the modified method (Schultz et al., 2000). They were detected through SDS-PAGE gel electrophoresis with β-GlcY reagent and Coomassie Brilliant Blue staining (Poon et al., 2012). The results showed the red smeared bands stained with β-GlcY reagent in each lane (**Figures S1A–C, G–I**). Low mobility of these stained bands was resulted from heavily glycosylated proteins, which was consistent with previous research results (Mashiguchi et al., 2004). No obvious bands were found in Coomassie Brilliant Blue staining (**Figures S1D–F, J–L**), which was because the polysaccharides covered the protein backbones (Sun et al., 2005).

### Identification of HCPs by Mass Spectrometry

**Figure 1:**
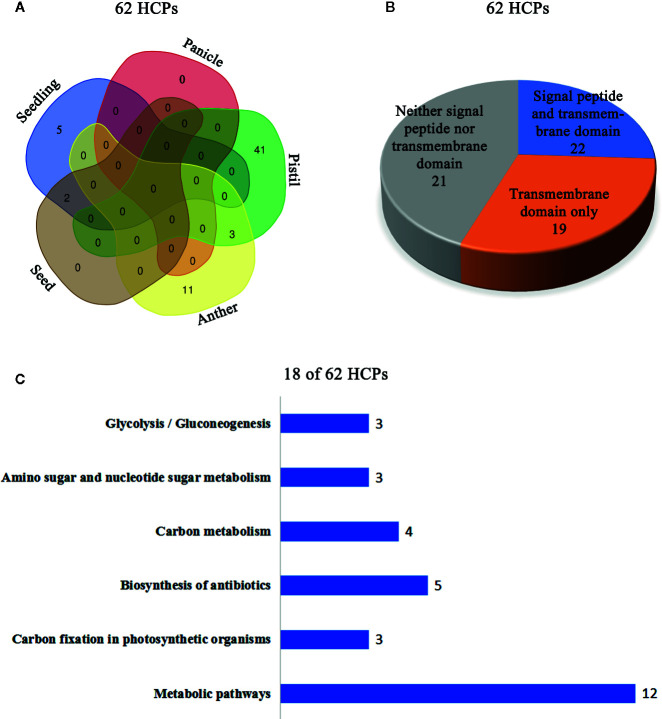
Cluster analysis of the 62 HCPs in rice. **(A)** The Venn diagram analysis of the 62 HCPs containing Hyps from various tissues and organs. **(B)** Signal peptide and transmembrane domain analysis of the 62 HCPs. **(C)** The 18 of the 62 HCPs containing Hyps are involved in different pathways.

The isolated glycoproteins were deglycosylated by the TFMS (Trifluoromethanesulfonic) acid, and then identified by mass spectrometry technique after digesting with trypsin. The 62 novel hydroxyproline-containing proteins were identified from the rice protein database (UniProt Knowledgebase) and shown with Venn diagrams (http://bioinformatics.psb.ugent.be/webtools/Venn/) (**Figure 1A**; **Table 1**, **Tables S1** and **S2**). The signal peptides and transmembrane domains were analyzed (**Figure 1B**; **Table 2**, **Table S3**) to confirm whether the 62 HCPs are likely to be glycosylated with arabinogalactan glycans in the secretory pathway. Among the 62 HCPs, the 22 HCPs contained both signal peptides and transmembrane domains, the 19 HCPs only contained transmembrane domains, while the 21 HCPs contained neither. The motifs of (Xaa-Pro)_n_ and Xaa-Pro_n_ were detected in these 22 and 19 HCPs (**Table 2**; **Table S7**), and of which the 5 and 7 HCPs contained more than 3 or more motifs, respectively. These motifs are considered to be possible sites for glycosylation (Shpak et al., 1999). Therefore, these 41 HCPs, especially the 12 HCPs containing 3 or more motifs were likely to be glycosylated with arabinogalactan glycan in the secretory pathway. The Hyps in these HCPs might be linked to glycans or may play a regulatory role in biological function of plant proteins (Shpak et al., 1999; Hirsilä et al., 2003). The pathway cluster analysis also indicated that the 18 of 62 HCPs were involved in various pathways and had different functions, while other 44 HCPs did not have enough annotations (**Figure 1C**).

**Table 1:** The 62 HCPs from different tissues and organs of rice identified by LC-MS/MS analysis.

Organ	Seedling	Panicle	Anther	Pistil	Seed	Total
**Total HCPs**	7	0	14	44	2	**62** (5)
**Specific HCPs**	5	0	11	41	0	**57**

The numbers in parentheses represent the numbers of proteins identified in more than one tissue or organ.

Specific HCPs: The proteins identified only in one tissue or organ.

**Table 2:** Signal peptide, transmembrane domain and motif analysis of rice HCPs.

HCPs with signal peptide and transmembrane domain		HCPs with transmembrane domain		HCPs without signal peptide and transmembrane domain		Total	
Total	≧3 motifs	Total	≧3 motifs	Total	≧3 motifs	Total	≧3 motifs
22	5	19	7	21	10	62	22

In this study, a total of 62 HCPs (**Table 1**, **Table S1**) containing the 114 Hyp sites (**Table S2**) were identified, and the 57 HCPs were specifically or predominantly expressed in different tissues and organs of rice, which implied that HCPs were essential for the growth and development of rice.

### The Characteristic Glycomodules of Hydroxyproline-Containing Proteins

**Figure 2:**
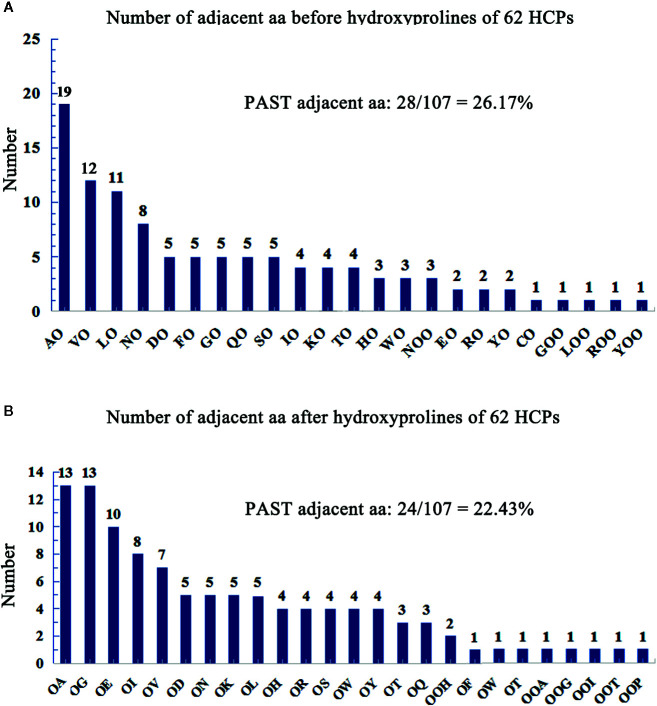
Number of adjacent aa before/after hydroxyprolines of 62 HCPs in rice. **(A)** Number of adjacent aa before hydroxyprolines of 62 HCPs. **(B)** Number of adjacent aa after hydroxyprolines of the 62 HCPs. PAST adjacent aa (amino acid): The aa (P, A, S, T) adjacent to O (Hyp) or OO (Hyp Hyp) on the upstream or downstream.

Hydroxyproline residues in the HCPs may form O-linked glycosylation modification (Showalter et al., 2010; Hijazi et al., 2012; Showalter et al., 2016) and play roles in plants (Hirsilä et al., 2003; Duruflé et al., 2017). AGPs containing Hyps are considered to have AG-type glycomodules (Ma et al., 2017). The number of adjacent amino acids to Hyps of AGPs was counted on the upstream or downstream (**Tables S4** and **S5**), and showed that the glycomodules of AO, OA, OG, VO, LO, and OE (O, hydroxylated proline) were abundant (**Figures 2A, B**). Previous studies display that the AGPs featured the presence of AO, OA, TO, VO, GO, and SO repeats distribute throughout the protein backbones (Schultz et al., 2002; Johnson et al., 2017). This feature of AGPs is widely used in bioinformatic identification of new AGPs. Similarly, the protein backbone of AGPs is rich in the amino acids of PAST (Ma and Zhao, 2010; Ma et al., 2017). The percentages of PAST adjacent to Hyps (O, OO, and OOO) on the upstream or downstream in the 62 HCPs, were 26.17% and 22.43%, respectively (**Figures 2A, B**). Interestingly, the glycomodules of AO, OA, OG, VO, LO, and OE were distributed abundantly, which might be used as references for the subsequent identification of new HCPs using bioinformatics. Moreover, the numbers of AO and OA glycomodules were significantly higher than others in the 62 HCPs (**Figures 2A, B**). It was similar to the AGPs feature that non-contiguous proline residues (e.g., APAPAP) were hydroxylated and glycosylated with arabinogalactan (AG) polysaccharides (Showalter et al., 2016).

### Hydroxyproline Sites of HCPs Without Signal Peptides and Transmembrane Domains

**Figure 3:**
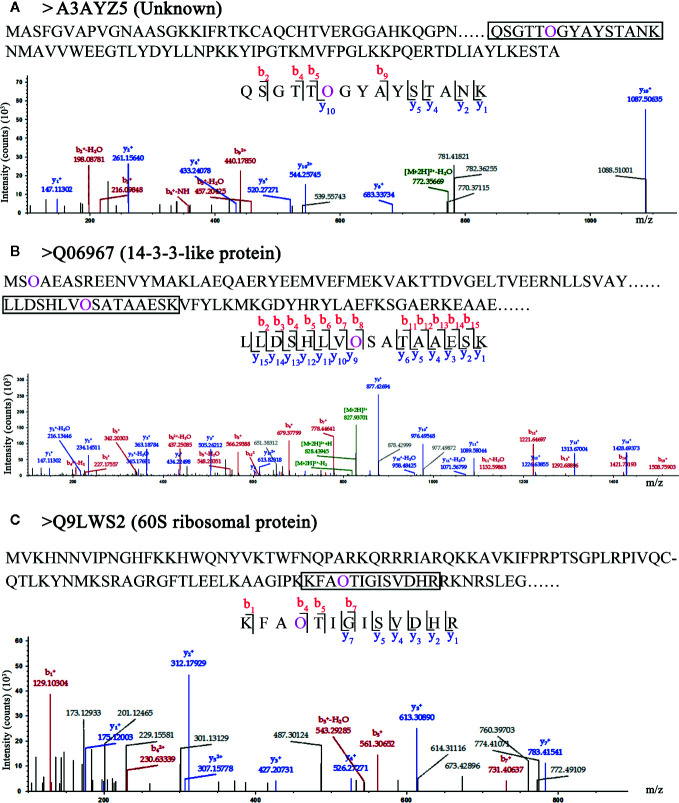
LC-MS/MS analysis of Hyp sites of 3 HCPs without signal peptide and transmembrane domain. Peptide fragmentation MS/MS spectra of **(A)** A3AYZ5 (Unknown), **(B)** Q06967 (14-3-3-like protein), and **(C)** Q9LWS2 (60S ribosomal protein) without signal peptide and transmembrane domain are shown for Hyp sites. Amino acid sequences labeled purple show the Hyp (O) sites of HCPs. The black box shows one of peptides containing Hyps and the peptide maps are also shown below. The peptide information was shown in **Table S6**.

In this study, the 62 HCPs with 114 hydroxyproline sites were identified in rice (**Table S2**), of which 21 HCPs contained neither signal peptides nor transmembrane domains. These proteins might be localized in the cytoplasm and/or non-secretory organelle, and were unlikely to be glycosylated with arabinogalactan glycans in the secretory pathway. An uncharacterized protein (A3AYZ5 in **Figure 3A**) contained one Hyp site and its function in rice was unclear. The 14-3-3 protein (Q06967 in **Figure 3B**) contained two Hyp sites, and one of the sites was located in 14-3-3 protein domain. The 60S ribosomal protein (Q9LWS2 in **Figure 3C**) contained one Hyp site. The three proteins without signal peptides and transmembrane domains contained Hyps, suggesting that these Hyps might play an important role in the function of rice proteins instead of glycosylating with arabinogalactan glycans.

### Hydroxyproline Sites of HCPs With Transmembrane Domains

**Figure 4:**
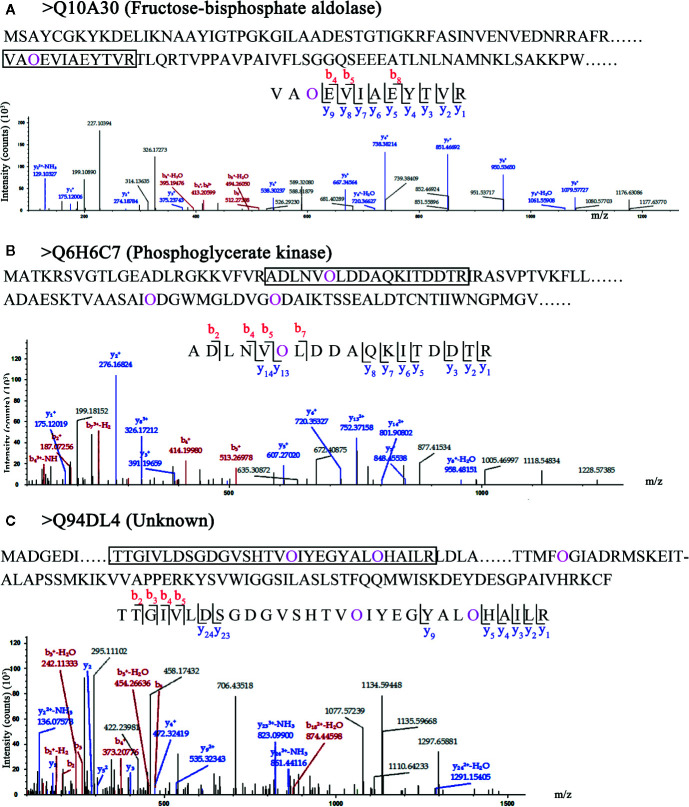
LC-MS/MS analysis of Hyp sites of 3 HCPs with transmembrane domain. Peptide fragmentation MS/MS spectra of **(A)** Q10A30 (Fructose-bisphosphate aldolase), **(B)** Q6H6C7 (Phosphoglycerate kinase), and **(C)** Q94DL4 (Unknown) with transmembrane domain are shown for Hyp sites. Amino acid sequences labeled purple show the Hyp (O) sites of HCPs. The black box shows one of peptides containing Hyps and the peptide maps are also shown below. The peptide information was shown in **Table S6**.

In the 62 HCPs, the 19 HCPs with transmembrane domains were identified, but they did not contain signal peptides. Although the 19 HCPs were not involved in secretory pathway, they were likely to be glycosylated with many motifs. The fructose-bisphosphate aldolase (Q10A30 in **Figure 4A**) with one Hyp site was identified and the site was located in the fructose-bisphosphate aldolase domain, indicating that the hydroxylation of proline might be involved in the function of fructose-bisphosphate aldolase. In the 19 HCPs, three proteins were identified with multiple Hyps. Two identified proteins, phosphoglycerate kinase (Q6H6C7 in **Figure 4B**) and the protein of unknown function (Q94DL4 in **Figure 4C**), were found to have three Hyp sites each. Although, based on current research, it is unclear whether proteins with multiple Hyps can be glycosylated, it is possible that glycans may be attached to certain Hyps.

### Hydroxyproline Sites of AGPs

**Figure 5:**
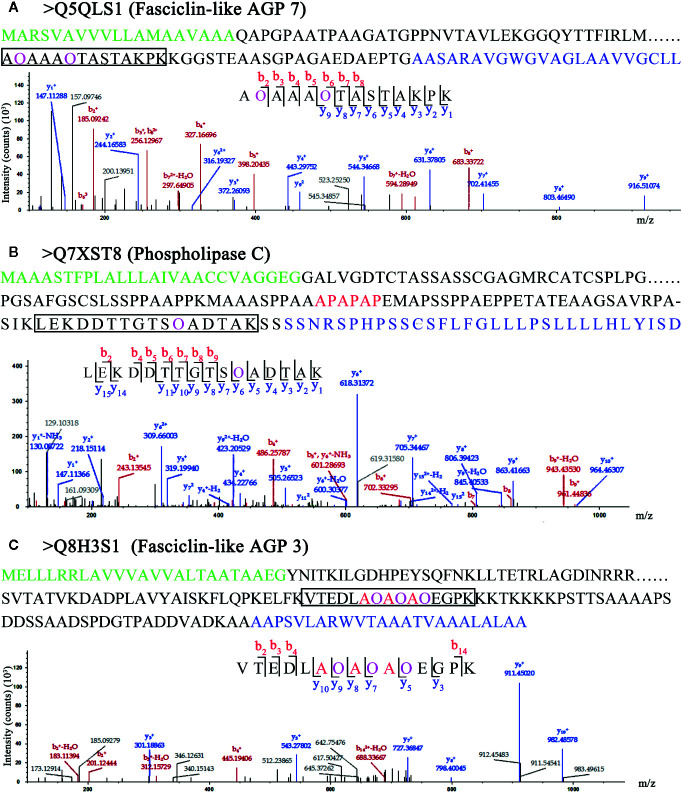
LC-MS/MS analysis of Hyp sites of 3 AGPs. Peptide fragmentation MS/MS spectra of **(A)** Q5QLS1 (Fasciclin-like AGP 7), **(B)** Q7XST8 (Phospholipase C), and **(C)** Q8H3S1 (Fasciclin-like AGP 3) are shown for Hyp sites. Amino acid sequences labeled green in **(A–C)** are signal peptides; blue in **(A–C)** are GPI anchor sequences. Amino acid sequences labeled purple show the Hyp (O) sites of HCPs. The black box shows one of peptides containing Hyps and the peptide maps are also shown below. The peptide information was shown in **Table S6**.

Chimeric AGPs are intrinsically disordered and important subfamilies of AGPs. Chimeric AGPs with Hyps were identified in rice, for example, Fasciclin-like AGP 7 (Q5QLS1 in **Figure 5A**), a chimeric AGP (Q7XST8 in **Figure 5B**) and Fasciclin-like AGP 3 (Q8H3S1 in **Figure 5C**). They all contained signal peptides, transmembrane domain, GPI anchor sequence, and different characteristic domains, which implied their various functions in rice. The Hyps were all located in the AGP-like region, which indicated that the hydroxylated region of proline residues are ubiquitous in AGPs. Furthermore, in both Q7XST8 (**Figure 5B**) and Q8H3S1 (**Figure 5C**), the APAPAP sequences that were considered to be characteristic glycomodule were detected. Surprisingly, three proline residues with hydroxylation in APAPAP sequences of Q8H3S1 were obtained. Previous studies indicate that the non-continuous hydroxyproline is usually linked to β-1,3-galactan main chain and β-1,6-galactan side chains (Shpak et al., 1999; Zhao et al., 2002; Xu et al., 2008). Therefore, the FLA3 might link to Arabinogalactan glycans in the site of APAPAP sequence.

### Distribution of AGPs in Vegetative Tissues and Organs of Rice

**Figure 6:**
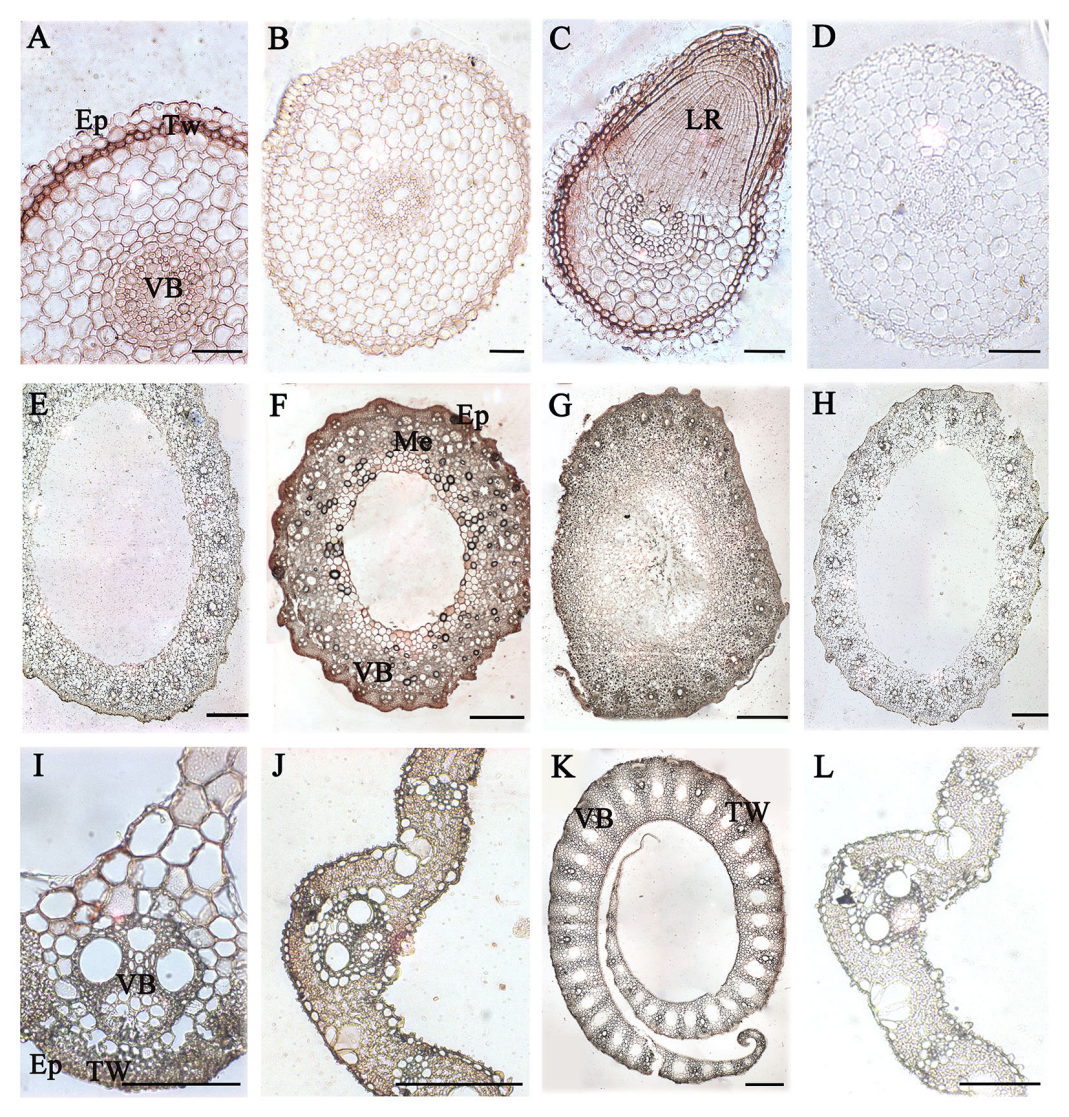
Distribution of AGPs in vegetative organs of rice. **(A)** 14 DAG root and **(B)** 60 DAG root. AGPs expressed in thick-walled tissues and vascular bundles under the epidermis. **(C)** Lateral root at 60 DAG. **(E)** 60 DAG stem and **(F)** 90 DAG stem. AGPs weakly expressed in mechanical tissues and vascular bundles under the epidermis. **(G)** Node at 60 DAG. **(I)** 60 DAG leaf and **(J)** 90 DAG leaf. AGPs were expressed throughout the tissues, including vascular bundles of epidermis and thick-walled tissues. **(K)** Leaf sheath at 90 DAG. No staining was detected in the control group **(D, H, L)**. Ep, epidermis. TW, thick-walled tissue. VB, vascular bundle. LR, lateral root. Me, mechanical tissues. Scale bars: 0.05 mm **(A, C**, **I)**, 0.1 mm **(B, D, K)** and 0.5 mm **(E–G, H, J, L)**. At least three independent biological and technical replicates were made.

Immunoenzyme localization of vegetative tissues and organs in rice different development stage was carried out with JIM13 antibody. The JIM13 antibody was derived from the embryonic suspension cells of carrot and bound to the β-D-GlcpA-(1-3)-α-d-GalpA-(1-2)-l-Rha epitope (Knox et al., 1991; Yates et al., 1996). In rice root, AGPs expressed in the epidermis, thick-walled tissues, and vascular bundles in the 14 DAG roots (**Figure 6A**) compared to the control group (**Figure 6D**), while significantly lower in 60 DAG roots (**Figure 6B**). The expression signals of AGPs were high in the site of lateral root formation (**Figure 6C**). In the 90 DAG stem (**Figure 6F**), the expression signals of AGPs were higher than that of 60 DAG (**Figure 6E**) compared to the control group (**Figure 6H**), and mainly distributed in thick-walled tissues and vascular bundles. In the stem nodes, the expression of AGPs mainly concentrated on the vascular bundles (**Figure 6G**). In the 60 and 90 DAG leaves and leaf sheathes, the signals of AGPs distributed throughout the tissues of the leaves, including vascular epidermis, mesophylls and veins (**Figures 6I–L**).

### Distribution of AGPs in Rice Anther and Pollen

**Figure 7:**
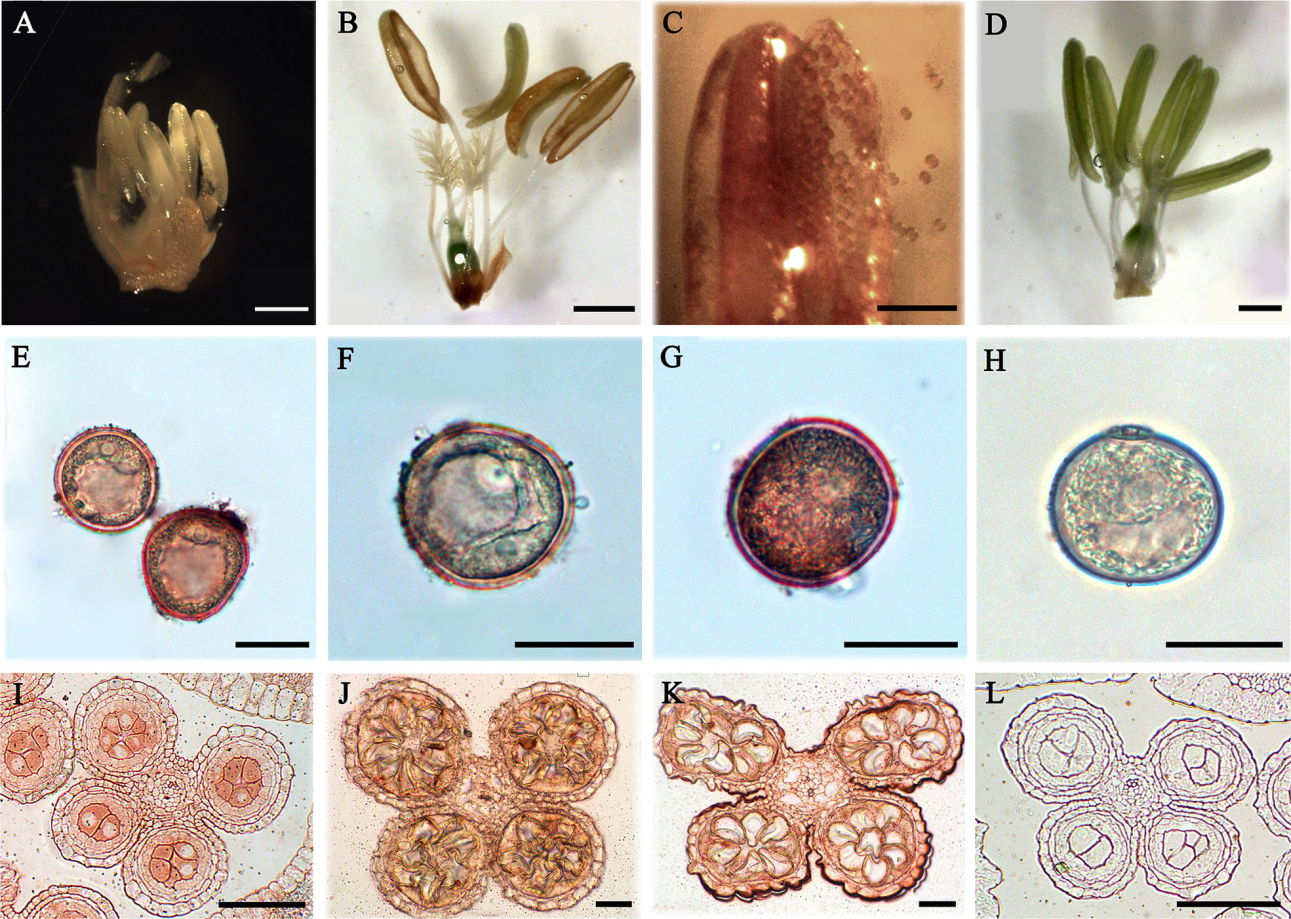
Distribution of AGPs in rice anthers and pollens. **(A–H)** Distribution of AGPs by β-GlcY staining. **(I–L)** Distribution of AGPs by β-immunoenzymatic staining. **(A)** Anther during pollen mother cell stage (containing pistil). **(B)** Anther of mature pollen stage (containing pistil). There was no staining in anther wall, but strong staining on the pollen of cracked anther **(C)**. **(E)** Pollen of late microspore stage. **(F)** Early dinuclear pollen. **(G)** Late dinuclear pollen. **(I)** Anther in microspore mother cell stage. **(J)** Anther in microspore stage. **(K)** Anther in early dinuclear pollen stage. No staining was detected in the control group **(D, H, L)**. Scale bars: 2 mm **(A)**, 1 mm **(B–D)**, 0.5 mm **(E–H)** and 0.05 mm **(I–L)**. At least three independent biological and three technical replicates were made.

In order to explore the expression of AGPs, anthers in different developmental stages of rice were detected with β-GlcY staining and immunoenzyme localization technique. The results showed that no obvious β-GlcY staining was detected in the anthers at pollen mother cell stage (**Figure 7A**). However, there was strong staining signal in the cracked mature anthers and the scattered pollens (**Figures 7B, C**) compared to the control group (**Figure 7D**). Furthermore, the AGPs of pollens were detected with JIM13 antibody and immunoenzyme technique. The results showed that strong expression signals of AGPs were distributed in the mononuclear, binuclear, and trinuclear pollens, especially in the pollen walls (**Figures 7E–H**). Furthermore, immunoenzyme localization of anthers at three different developmental stages was performed with JIM13 antibody combined with semi-thin sectioning technique. In the pollen mother cell, mononuclear and binuclear stage pollens, strong AGPs immune signal was detected in the whole anthers (**Figures 7I–L**).

### Distribution of AGPs in Rice Embryos

**Figure 8:**
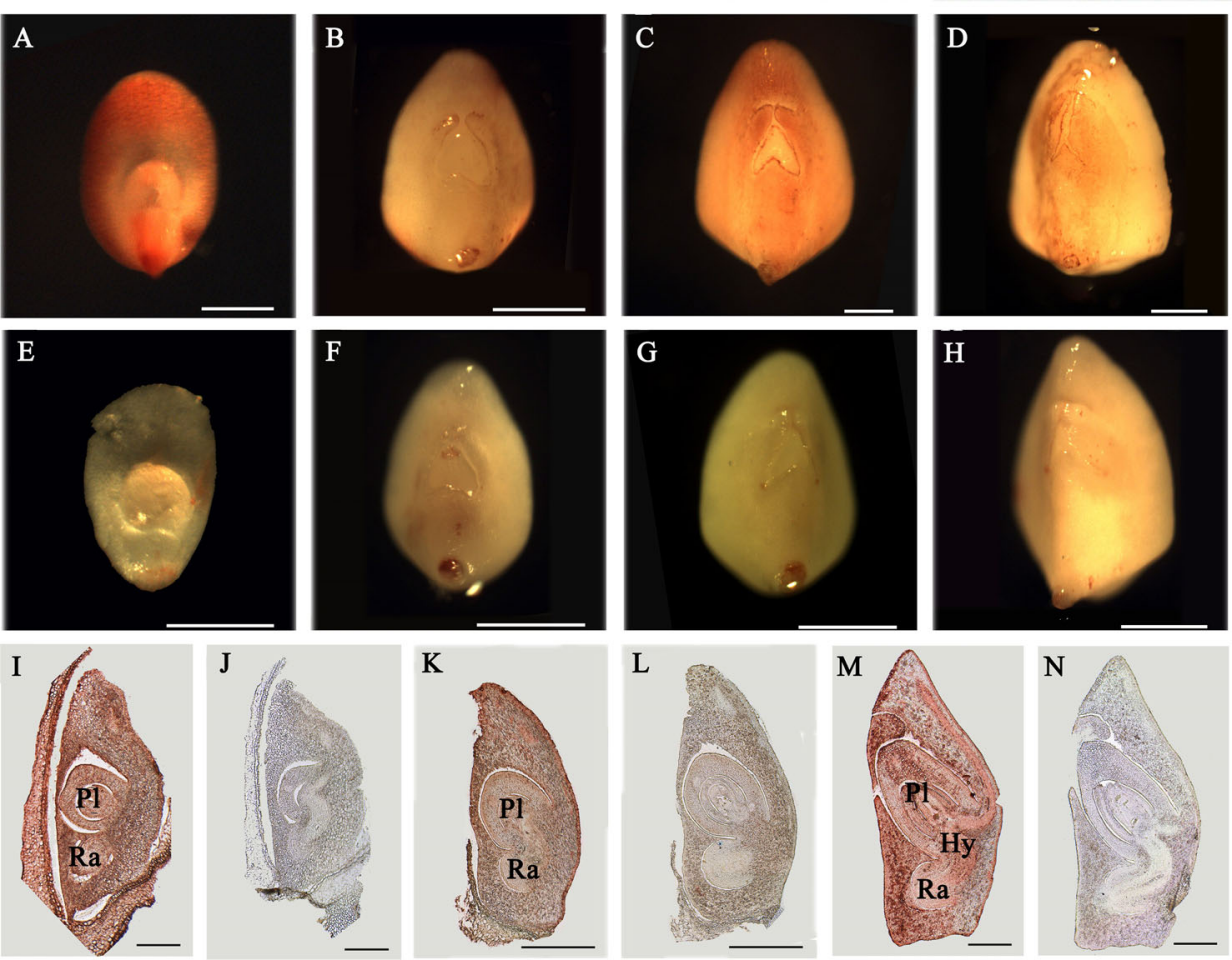
Distribution of AGPs in rice embryos. **(A–H)** Distribution of AGPs by β-GlcY staining in rice embryos. **(I–N)** Immunoenzyme localization of AGPs in rice embryos. **(A)** 5 DAP embryo. **(B)** 6 DAP embryo. **(C)** 8 DAP embryo. **(D)** 10 DAP embryo. **(I)** 8 DAP embryo. **(K)** 10 DAP embryo. (M) 15 DAP embryo. No staining was detected in the control group **(E–H, J, L, N)**. Ra, radicle. Pl, plumule. Hy, hypocotyl. Scale bars: 0.25 mm **(A–H)** and 0.5 mm **(I–N)**. At least three independent biological and three technical replicates were made.

In order to detect the expression of AGPs in rice flower and seed development, the total protein of inflorescences (10.5 cm and 27 cm) and seeds (3–5 DAP and 20 DAP) were extracted. The results showed that the bands of AGPs ranged from 31.8 to 223 kDa (**Figure S2**). The dispersive protein hybridization bands were caused by the various glycosylation of AGPs. Furthermore, the distribution of AGPs was detected with β-GlcY reagent in different developmental stage of embryos (5, 6, 8 and 10 DAP). The strong AGPs immune signal was located in the coleoptile primordium and the top and back parts of 5 DAP embryos (**Figure 8A**). The embryo at 5 DAG is the critical period for the initiation of differentiation of rice embryo organs, which suggested AGPs played an important role in early embryonic differentiation of rice. As the embryos continue to develop, the immune signal of AGPs was weakened in 6 DAP embryo (**Figure 8B**) and enhanced in 8 DAP embryo (**Figure 8C**), while decreased again in 10 DAP embryo (**Figure 8D**). At the same time, there was not immune signal of AGPs in the control groups (**Figures 8E–H**).

The expression of AGPs in internal tissues after rice embryo differentiation was detected with JIM13 antibody and paraffin sectioning technique. In 8 DAP embryo, AGPs expressed strongly throughout the embryo; including radicle, hypocotyl and plumule (**Figure 8I**), while weakly expressed in 10 DAP (**Figure 8K**). There was also strong expression signal of AGPs in 15 DAP embryos (**Figure 8M**). There was not immune signal of AGPs in the control groups (**Figures 8J, L, N**).

In conclusion, there was obvious AGP expression signal and change during the early embryo development of rice, which may be caused by the successive expression of different AGPs to maintain embryo development.

## Discussion

### AGPs and β-GlcY Reagent

Yariv phenylglycoside is a chemical reagent that was initially developed for precipitation of polysaccharides (Yariv et al., 1962; Yariv et al., 1967). Further research demonstrates that both β-glucosyl Yariv phenylglycoside (β-GlcY) and β-galactosyl Yariv phenylglycoside (β-GalY) can bind to AGPs, whereas α-glucosyl Yariv (α-GlcY) and α-galactosyl Yariv (α-GalY) cannot bind to AGPs (Nothnagel and Lyon, 1986; Kitazawa et al., 2013). Subsequently, it was reported that Yariv phenylglycosides can specifically bind β-1,3-galactan chains longer than five galactan residues (Kitazawa et al., 2013). The glycans of AGPs are type II polysaccharides with a typical 1,3-galactan main chain and 1,6-galactan side chains, to which L-Ara and other auxiliary sugars, such as GlcA, 4-O-methyl-GlcA, L-Fuc, L-Rha, and Xyl are attached (Ling et al., 2012; Kitazawa et al., 2013). Therefore, β-GlcY has been used in the extraction, staining, and processing of various tissues of plants to study the function of AGPs.

However, previous studies have also shown that the binding between β-GlcY reagent and AGPs is not completely specific, that is, some AGPs cannot bind to β-GlcY reagent; the other can bind to it but not AGPs (Lind et al., 1994; Serpe and Nothnagel, 1994; Nothnagel, 1997). The latest research results show that β-GlcY reagent binds to the β-1,3-galactan of AGPs (Kitazawa et al., 2013). It was suspected that β-GlcY reagent may not only bind β-1,3-galactan of AGPs, but also bind to β-1,3-galactan of other glycoproteins, which was the reason that some proteins that are not AGPs can also bind to reagent. At the same time, the AGPs that cannot bind to the reagent may not have a typical 1,3-galactan main chains, or the chains are short that there are not enough β-GlcY binding sites. Some AGPs were not identified, which was speculated that they might not be precipitated with β-GlcY reagent. Therefore, the 62 HCPs identified in this study were probably not all glycoproteins, but they were hydroxyproline-containing proteins (HCPs). Nevertheless, it is thought that AGPs can be bound to β-GlcY, and the reagent is still an indispensable for AGPs functional study (Serpe and Nothnagel, 1994).

### High Degree of Glycosylation Modification of HCPs

Previous studies have also found that glycosylation in AGPs can even account for more than 90% of the molecular weight, which indicates that the degree of glycosylation in AGPs was usually high (Sun et al., 2005). Our results showed the similar phenomenon that there was no obvious Coomassie Brilliant Blue staining of glycoproteins in different rice tissues and organs (**Figures S1D–F, J–L**), which may be caused by the fact that its glycosylation modification encapsulated the protein backbones and Coomassie Brilliant Blue cannot bind to the amino acids. Further β-GlcY staining revealed that AGPs usually have typical diffuse bands, which are mainly caused by the different degree of AGPs glycosylation.

Recently, the studies on glycosyltransferases revealed that they are essential for post-translational modification of AGPs and affect the growth and development of various tissues and organs in higher plants (Liang et al., 2010; Wu et al., 2010; Endo et al., 2013). For example, the study on *HPGT1/2*/*3* indicates that approximately 90% of the endogenous Hyp O-galactosylation activity is attributable to the loss-of-function of these three enzymes (Ogawa-Ohnishi and Matsubayashi, 2015), further illustrating that the glycosylation modification of AGPs is linked to the Hyps. However, little is known about how AGPs are added to glycosyl chains, which need further research to reveal.

### Different Hydroxylation and Glycosylation in Various Subfamilies of HCPs

There are two main types of glycosylation in plants, including N-glycosylation and O-glycosylation. The N-glycosylation is characterized well and occurs on asparagine residues, whereas O-glycosylation, which is the glycosylation of hydroxyl groups on the side chains of amino acids, is not well characterized. In yeast, fungi and animals, O-glycosylation binds to serine and threonine residues, while O-glycosylation in higher plants is more complex than in these organisms and usually links to Hyp residues apart from serine and threonine residues (Shimizu et al., 2005), for example, the AGPs. A previous study demonstrates that non-contiguous Hyp residues are the sites for binding to arabinogalactans (Kieliszewski and Shpak, 2001). Further research showed that the glycomodules of AP, PA, TP, VP, GP, and SP are the characteristic repeats distributed throughout the AGP protein backbones, which are often used as the reference for the identification of AGPs in bioinformatic analysis (Schultz et al., 2002; Johnson et al., 2017). In this study, it was found that the glycomodules of AO and AO were abundant in AGPs. Moreover, the results showed that the prolines were hydroxylated among these glycomodules, and the glycomodules of AO, OA, OG, VO, LO, and OE were rich in HCPs (**Figures 2A, B**), especially AO and OA, which indicated alanine residues were important for hydroxylation of proline residues. The Hyp sites analysis indicated that most Hyps located in AGP-like region (**Figures 5A–C**). However, Hyps might not be fully identified, it was because too little glycoproteins precipitated with β-GlcY reagent.

The studies indicate that continuous hydroxyproline is often highly arabinosylated, while non-continuous hydroxyproline is usually linked to β-1,3-galactan main chain and β-1,6-galactan side chains (Shpak et al., 1999; Zhao et al., 2002; Xu et al., 2008). AGPs contain characteristic glycomodules, including AP, PA, SP, TP, VP, and GP (Tan et al., 2003; Johnson et al., 2017). Our results also showed that the glycomodules of AO, LO, VO, OE, OG, and OA (**Figures 2A, B**) were more abundant, which was consistent with previous studies. The previous studies have identified seed storage proteins in rice (Kishimoto et al., 1999), but these proteins were not in our data set; it might be because the mature seeds of rice did not contained in our extraction of proteins.

In this study, AGPs with Hyps were identified, and these hydroxylation sites of proline residues were likely to be the sites to link to Arabinogalactan glycans (**Figures 5A–C**). The 22 HCPs with signal peptides and transmembrane domains and 19 HCPs with transmembrane domains were presented in the 62 HCPs (**Figure 1B**; **Table 2**, **Table S3**). The 12 of them contained 3 or more motifs, and might be the candidates for glycoprotein. Continuous hydroxylated proline residues were likely to be highly arabinosylated. There were 21 HCPs identified without signal peptides and transmembrane domains, and these proteins may localized in the cytoplasm or non-secretory organelle. There are few reports describing the presence of Hyp-containing cytoplasmic proteins. There were 10 HCPs containing 3 or more motifs (**Figure 1B**; **Table 2**, **Table S3**), it may be the candidate for linking to glycans. There were five actin proteins and seven ribosomal proteins identified in this study, but few motifs in these proteins were detected. The hydroxylation of these proteins might be related to the structure and function rather than glycosylation. In our study, the hydroxylation of proline residues in rice is ubiquitous, indicating that hydroxylation can be linked to glycosylation modification and involved in the regulation of various functions in plants.

### Evolutionarily Conservation of AGPs in Plant Kingdom

AGPs are a class of prevalent glycoproteins in plant kingdom. It has been found that there are high sequence similarities and evolutionary conservation in different monocotyledonous and dicotyledonous plants. A total of 7216 putative AGPs were identified from 47 selected plant species, and classical AGPs, FLA, PAG, and XYLP could be found in all selected species except that classical AGPs and XYLP were absent in *Chlamydomonas reinhardtii* (Ma et al., 2017). There are also great similarities in expression and function of AGPs. For example, *AtAGP6* and *AtAGP11* specifically express in anthers of *Arabidopsis*, and play an important role in pollen development (Levitin et al., 2008; Coimbra et al., 2009; Coimbra et al., 2010). Their homologous genes, *BcMF8* and *BcMF18*, have the similar expression and function (Lin et al., 2014; Lin et al., 2018). It was thought that the glycosylation of AGPs in different species have great similarity, but few studies have been conducted about the structural composition and biological functions of glycoproteins in AGPs.

### Functional Redundancy of AGPs

The identification of AGPs is based on the composition of its glycosylation modification, and there are different functional domains, which lead to higher heterogeneity and functional diversity. AGPs are essential in tissue and organ development (Huang L. et al., 2008; Bossy et al., 2009; MacMillan et al., 2010; Tryfona et al., 2012; Lin et al., 2018), embryogenesis (Pan et al., 2011; Poon et al., 2012; Steinmacher et al., 2012), plant resistance (Shi et al., 2002; Huang G. Q. et al., 2008; Mashiguchi et al., 2008; Gong et al., 2012), hormone regulation (Park et al., 2003; Liu and Mehdy, 2007), bacterial infection (Gaspar et al., 2004; Nguema-Ona et al., 2013), and double fertilization (Coimbra et al., 2008; Lee et al., 2008).

AGPs have functional redundancy in various plants. For example, no abnormal phenotype was found in the single gene mutant of *AtENODL11/12/13/14*/*15* in *Arabidopsis*, while obvious seed abortion was observed in the mutation of all five genes, which indicates that the *Arabidopsis* AGP family does have functional redundancy (Hou et al., 2016). However, in *Arabidopsis*, single gene mutants also appear obvious plant growth and development abnormalities. For example, the single gene knockdown of *AtFLA3* could results in seed abortion (Li et al., 2010), although *AtFLA5* and *AtFLA14* are homologous genes to *AtFLA*3. Bioinformatic identification and evolution analysis of AGPs illustrate that there are 282 AGPs in rice more than the 151 in *Arabidopsis* (Ma et al., 2017), which indicates that AGPs maybe have more functional redundancy in rice. The increased number of AGPs may help to better adapt to the changing biological environment to maintain the competitiveness of the rice itself.

## Conclusions

The HCPs play vital roles during plant growth and development, but the mutants of HCPs are difficult to obtain in rice. The birth of CRISPR/Cas9 technique solves this problem, and this technique is even more efficient for gene editing in rice than in *Arabidopsis*. The functional research of HCPs is also affected by glycosylation modification, the protein antibody is difficult to obtain, and the protein glycosylation modification is difficult to investigate. Recently, more powerful mass spectrometry technique may provide effective research tools for identifying HCP glycosylation modification sites, and more advanced cryo-electron microscopy technology (Hochscherf et al., 2018) has made it possible to explore the crystal structure of HCPs, especially the structure of glycosylation, which open a new insight for the future research of HCPs.

In this study, a total of 62 HCPs and 114 hydroxylation sites of proline residues in rice were identified. In the 62 HCPs, the glycomodules of AO, OA, OG, VO, LO, and OE were abundant. There were 22 HCPs with both signal peptides and transmembrane domains, and 19 HCPs only with transmembrane domains, while 21 HCPs without signal peptides and transmembrane domains. AGPs in rice were identified with Hyps and might be glycosylated with arabinogalactan glycan. In addition, it was found that AGPs were extensively expressed in different tissues and organs detected by using β-GlcY reagent and JIM13 antibody. This work might lay the foundation for further studying the functions of HCPs and hydroxylation of prolines in rice.

## Data Availability Statement

The proteomic data were submitted into iProX (http://www.iprox.org) (Ma J. et al., 2019) and the project ID1 is IPX0001999000.

## Author Contributions

JZ designed the research plans, guided the whole study, and wrote and modified the paper. RL performed most of the experiments, analyzed the research results and wrote the paper; LY performed bioinformatics analysis and analyzed the data; FD performed localization of AGPs with JIM13 antibody and β-GlcY reagent. XZ analyzed the part of mass spectrometry data.

## Funding

This research was supported by the National Science Foundation of China (31870303, 31670312, and 31370348).

## Conflict of Interest

The authors declare that the research was conducted in the absence of any commercial or financial relationships that could be construed as a potential conflict of interest.
