# Supplementary material for: Identification of Hydroxyproline-Containing Proteins and Hydroxylation of Proline Residues in Rice
Source: Front Plant Sci. 2020 Aug 7;11:1207. doi: 10.3389/fpls.2020.01207 (PMC7427127; doi:10.3389/fpls.2020.01207)
Supplement: Supplementary file 1 [file DataSheet_1.docx]

Supplementary Material

# Supplementary Figure Legends

**Figure S1 Detection of proteins in different tissues and organs of rice by electrophoresis.**

(A-C and G-I) Rice proteins with β-GlcY staining. (D-F and J-L) Rice proteins with Coomassie Brilliant Blue staining. Ro, 14 DAG root. Sh, 14 DAG shoot. Pa, P1-P6 panicle. An, mature anther. Pi, mature pistil. Se, 1-10 DAP seed. M, protein marker. AG, arabic gum. There were two independent biological replicates of each tissue or organ.

**Figure S2 Western blot of AGPs in rice inflorescences and seeds.**

The AGPs expression with western blot by JIM13 antibody in 10.5 cm and 27 cm inflorescences, and 3-5 DAP and 20 DAP seeds. At least three independent biological and three technical replicates were made.

**Supplementary Tables.**

**Table S1 The 62 HCPs containing hydroxyprolines identified by mass spectrometry in rice.**

**Table S2 The 114 Hyps identified in the 62 HCPs of rice.**

**Table S3 Signal peptide and transmembrane domain analysis of the 62 HCPs in rice.**

**Table S4 The adjacent aa sequences of hydroxyprolines in the 62 HCPs of rice.**

**Table S5 The number of adjacent aa of hydroxyprolines in the 62 HCPs of rice.**

**Table S6 The information of peptides in Figure 4-6.**

**Table S7 Analysis of motifs in the 62 HCPs of rice.**
