# Supplementary figures and images for: Identification of Hydroxyproline-Containing Proteins and Hydroxylation of Proline Residues in Rice
Source: Front Plant Sci. 2020 Aug 7;11:1207. doi: 10.3389/fpls.2020.01207 (PMC7427127; doi:10.3389/fpls.2020.01207)

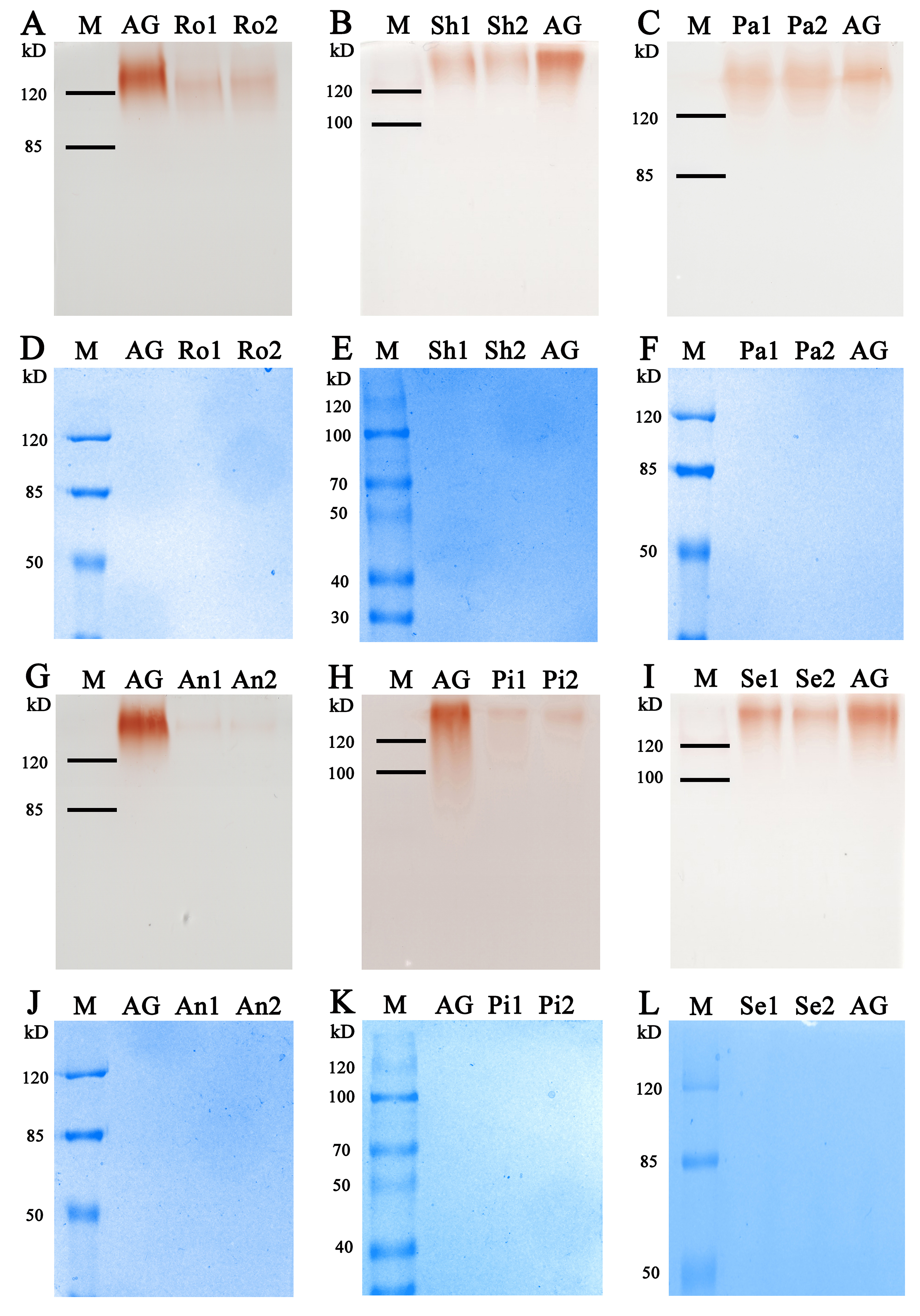

Supplement: Supplementary file 2 [file Image_1.jpeg]

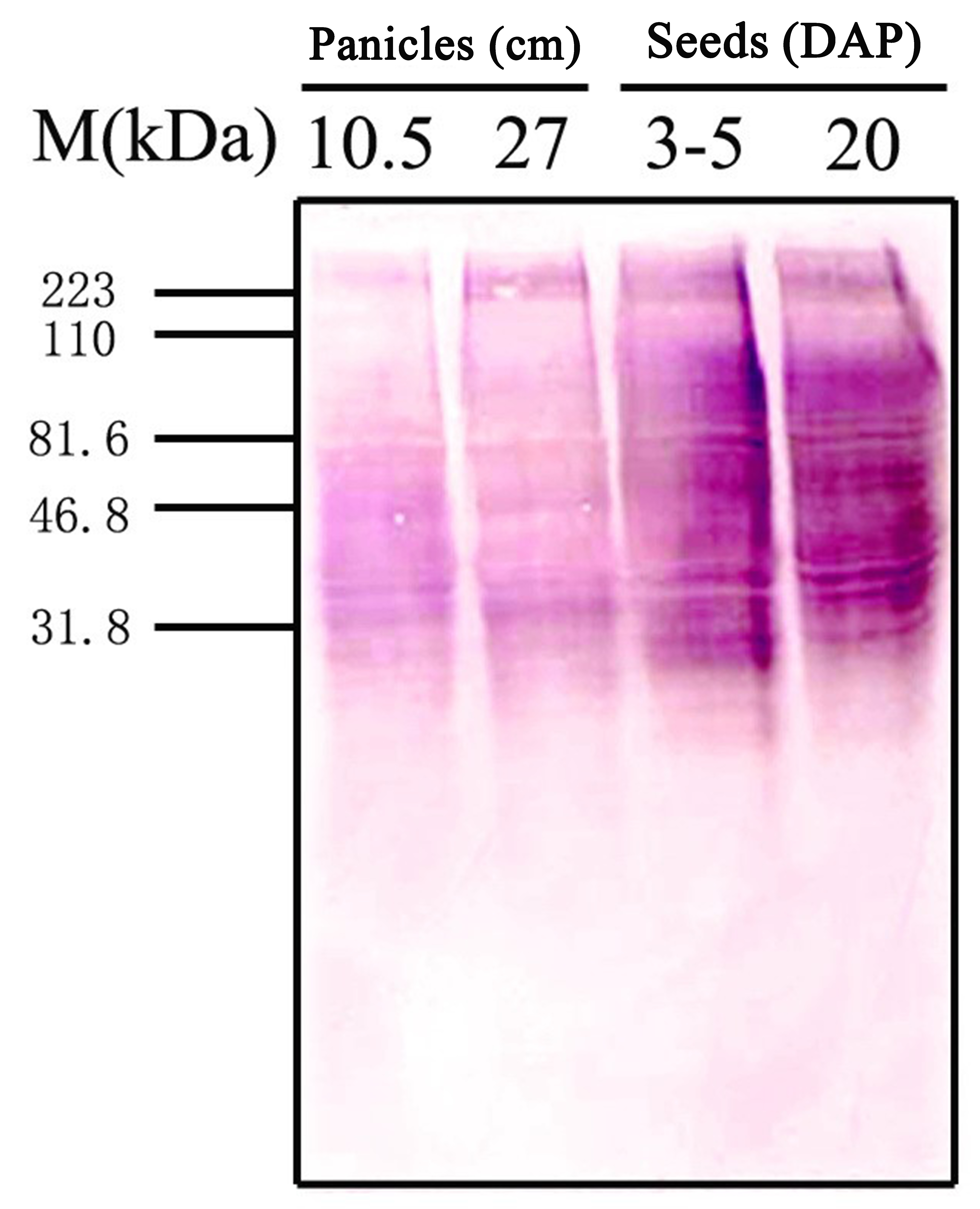

Supplement: Supplementary file 3 [file Image_2.jpeg]
